# Quality of guidelines for infection management in sepsis: a critical appraisal using the AGREE II instrument

**DOI:** 10.1186/s12874-025-02491-8

**Published:** 2025-02-21

**Authors:** Guo-Xun Yang, Ting Que, Yi-fei Wang, Xiao-Bo Liu, Shu-Qian Dou, Shi-Ling Pu, Xin Wang, Kong-Jia Wu, Yuan Wang, Qi Wang, Wen-Jun Liu

**Affiliations:** 1 The Second Affiliated Hospital of Kunming Medical University, Kunming Medical University, Kunming, 650101 Yunnan China; 2 The First Affiliated Hospital of Dali University, Dali, Yunnan 671000 China; 3 The Research Center of Burn in Yunnan Province, Kunming, Yunnan 650101 China

**Keywords:** Sepsis, Infection management, Guidelines, Quality assessment, AGREE II

## Abstract

**Objectives:**

The aim of this study was to systematically assess the methodological quality of current sepsis infection management guidelines and identify gaps in knowledge that limit evidence-based practice.

**Methods:**

A systematic search was conducted to obtain guidelines for the management of sepsis infections (2012–2021), and three reviewers independently assessed the quality of eligible guidelines using Appraisal of Guidelines for Research and Evaluation (AGREE II) tool. The intraclass correlation coefficients (ICCs) were used to measure the agreement between reviewers. The Grading of Recommendations, Assessment, Development and Evaluation (GRADE) approach was used to analyze the strength of recommendation and level of evidence of the guideline, and the number of recommendations, strength of recommendation, and level of evidence were determined.

**Results:**

Eleven guidelines for the management of sepsis infection were identified. An overall high agreement among the evaluators for each domain was observed (ICC ranged from 0.850 to 0.959). The overall scores of the included guidelines were all over 60% (range, 62.3-89.90%), which were worthy of recommendation for clinical use; among them, 4 guidelines had an overall score of over 80%, which were high-quality guideline articles. In terms of the quality domains of the guidelines, the scope and purpose domain and the clarity of expression domain had the highest average scores, which were 93.6% (range, 79.6–98.1%) and 91.4% (range, 64.8–98.1%), respectively, while the applicability domain had the lowest average score, which was 64.8% (range, 51.4–76.4%). The strength of the recommendations of the guideline recommendations was mainly weak, accounting for 73.4%; the level of evidence cited was mainly very low quality (60.2%) and low quality (28.1%).

**Conclusions:**

The quality of sepsis infection management guidelines varies, but the overall quality level is satisfactory. Improving the low-quality areas of sepsis guidelines, attempting to resolve existing problems and controversies, and improving the quality of research evidence will be effective ways for developers to upgrade sepsis guidelines.

**Supplementary Information:**

The online version contains supplementary material available at 10.1186/s12874-025-02491-8.

## Background

Sepsis is a major global health problem, with more than 40 million patients with sepsis and 11 million sepsis-related deaths recorded each year worldwide, with mortality estimates as high as 26.6% in hospital-treated sepsis and 41.9% in ICU-treated sepsis [1–3]. In addition, the cost of sepsis treatment is very high, and the use and cost of health care resources increase with increasing severity, causing a certain degree of impact on economic development [4–6]. For example, sepsis is the most common cause of in-hospital death in the United States. In 2017, the survey results showed that sepsis was the disease with the highest cost of hospital treatment, accounting for 8.8% of the total cost of all hospitalizations, costing more than $38 billion per year, and the above results increased compared with 2013 [7, 8]. In 2017, World Health Organization (WHO) member states proposed improving the identification, documentation, prevention and treatment of sepsis as a global health priority [9]. Although sepsis impacts health care systems across all age groups and countries and remains the most common cause of admission and death in intensive care units (ICUs) [10], we should recognize that sepsis is treatable and that timely implementation of targeted interventions can improve outcomes [11, 12].

Infection management is an extremely important part of the treatment of sepsis. At present, in order to guide the effective management of sepsis infection and improve the prognosis of sepsis patients, a large number of local, national and international expert organizations have formulated guidelines for the management of sepsis and septic shock [13–23]. Ideal sepsis management guidelines are to integrate all relevant research evidence, conduct systematic reviews and/or meta-analyses, arrive at the best research evidence results, and make the most advantageous recommendations based on evidence. In addition, clinicians and guideline users are able to clearly combine clinical management decisions for patients with sepsis when referring to the guidelines. However, there is a wide variety of published guidelines for the management of sepsis, some degree of heterogeneity between guideline recommendation programs, and uneven quality of cited research evidence. In addition, for the recommended items with high homogeneity between the guidelines, there are no relevant studies to summarize, sort out, analyze and report the high-quality study evidence cited in the guidelines as well as the high-quality study evidence not found by the guidelines. These somewhat limits clinical guideline users’ ability to refer to guidelines for clinical decision-making and does not maximize the role of guidelines in assisting clinical decision-making and improving health care systems.

Currently, there is still a lack of systematic assessment of existing guidelines related to the management of sepsis infection worldwide, and the distribution of the level of evidence on which recommendations for the management of infection in sepsis guidelines are based has not been clarified. In addition, some methodological quality issues in guidelines remain prevalent over time and remain poorly addressed. For these reasons, we used the AGREE II tool to evaluate methodological quality of sepsis infection management guidelines. On the one hand, we can identify prevalent quality issues after effective data assessment, provide effective methodological strategies for guideline development, inform guidelines on what information should be reported and how information should be reported, and identify knowledge gaps that limit evidence-based practice and highlight potential opportunities for improvement. On the other hand, we can grasp the overall quality of relevant sepsis guidelines, discover high-quality guidelines, and provide theoretical references for medical workers to conduct clinical practice, so as to better guide clinical practice and application.

## Methods

### Study design

The study was conducted according to the Preferred Reporting Items for Systematic Reviews and Meta-Analysis Protocols (PRISMA-P) [24]. The AGREE II instrument was used to comprehensively assess and analyze the quality of sepsis infection management guidelines.

### Literature search strategy

The following databases were systematically searched for this study: PubMed, Web of Science, Ovid, Science Direct, CNKI, and Wanfang Medical Database. Relevant official websites with possible sepsis guidelines were also searched: BMJ (https://www.bmj.com/), NICE (https://www.nice.org.uk/guidance/), NGC (https://www.guideline.gov/), SIGN (http://www.sign.ac.uk/), Google and Baidu. We limited the database search to 10 years (01 Jan 2012 to 31 Dec 2021), taking into account the update of medical knowledge and ensuring the comprehensiveness of medical knowledge as much as possible. The search terms included the following: “sepsis” (sepsis/ pyohemia/ pyaemia/ pyemia/ septicemia/ septic shock), “guideline” (guideline*/ statement*/ recommendation*/ consensus), and “guide words” (management/ treatment/ therapy/ diagnosis). Field identifiers searched were limited to the title. First, the subheading words under sepsis, guideline, and guide words were searched with the logical operator OR, respectively, and then calculated with the following search formula: “sepsis” AND “guideline” OR “guide words”. In addition, references included in the guidelines were reviewed and if relevant sepsis guideline articles were identified, a manual search was performed to ensure guideline recall.

### Guidelines’ selection

The inclusion criteria were as follows: (1) the latest sepsis guidelines; (2) guidelines with recommendations for sepsis infection management; (3) full text articles; and (4) published in both English and Chinese. The exclusion criteria were as follows: (1) older versions of guidelines; (2) duplicated guidelines; and (3) review, interpretation, or summary documents of sepsis guidelines. Two reviewers (G.X.Y and X.W) independently reviewed the literature search results in strict accordance with the inclusion and exclusion criteria, and in case of disagreement, they were handed over to a third party to intervene in a joint resolution to ensure the final accuracy rate of the included guidelines. After the guideline inclusion is completed, the basic information of the guideline is extracted.

### Quality evaluation of guidelines

The AGREE tool was developed to address variability in guideline quality and provided a framework for assessing the quality of guidelines, providing methodological strategies for developing guidelines, and informing activities such as what information should be reported in guidelines and how information should be reported. After continuous practice and improvement, the latest version, AGREE II (2017 version), has been released and has been widely used worldwide [25]. In this study, we used the latest version of the AGREEII instrument to assess the included sepsis infection management guidelines. According to AGREE II user manual, we scored the included guidelines in terms of six quality domains (Additional file 1).

We selected three clinical healthcare workers (G.X.Y, W.J.L, T.Q) with experience in clinical guideline assessment to independently assess the included guidelines. The above assessors needed to complete the training of AGREE on-line tutorials in advance and be familiar with and master the AGREE II user manual before performing blind scoring. Each item of the AGREE II instrument was assessed independently of other items, with few overlapping or crossing parts. Each item was scored on a 7-point scale: a score of 1 was strongly disagree and a score of 7 strongly agree. A score of 1 was assigned if there was no information related to the item, the concept was poorly reported, or the authors explicitly stated that the criteria were not met. A score of 2 to 6 was assigned if the report of the relevant item did not meet all criteria or considerations. Scoring was based on completeness and quality of reporting and increased as more criteria were met and considered for resolution. A score of 7 was assigned if the report was of excellent quality and met all criteria and considerations stated in the user manual. In addition, we would discuss items that differ by more than or equal to 3 points. Finally, the scores of the evaluators were presented to the statistician and each domain score was calculated using the following formula: (Obtained score – Minimum possible score) / (Maximum possible score – Minimum possible score) × 100%.

The domain scores were categorized as “high” (≥ 80%), “medium” (60-79%), “low” (40-59%), and “very low” (< 40%). We also needed to finally assess whether the included guidelines were recommended for clinical practice. The AGREE II panel divided the overall assessment into three categories: recommended, revised recommended, and not recommended. Because there was no reference standard for the meaning of scores in AGREE II user manual, we cited assessment methods that were practiced through multiple guidelines evaluating articles: final guideline scores > 60%, recommended; 30-60%, revised recommended; < 30%, not recommended [26–28].

### Strength of recommendation and level of evidence

Currently, there are many evidence and recommendation grading standards worldwide, and therefore, the grading methods used in different guidelines may vary. To identify major gaps between evidence and treatment, identify differences in codes expressing strength of recommendation and level of evidence across guidelines, and avoid confusion in applying recommendations because confusing codes to guideline users, we introduced GRADE criteria (Additional file 2) [29], a widely used recommendation and evidence grading system. The GRADE system classifies the strength of recommendation into two levels: “strong recommendation” and “weak recommendation”, and the quality of evidence into four levels: “high”, “medium”, “low”, and “very low.” In addition, the GRADE working group recommends that when the benefits and harms are uncertain and it is difficult to summarize the evidence using the GRADE method, it is appropriate to use best practice statements (BPS) [30]. In this study, we extracted the recommendation items in the guidelines and relevant research evidence, and then used the GRADE criteria for grading the strength of recommendation and level of evidence.

### Statistical analysis

We used a descriptive statistical analysis to calculate normalized scores for each domain, expressed as percentages, and presented the range and mean number for each domain. Two-way analysis of variance was used to calculate intraclass correlation coefficients (ICCs) to test whether the scores of the three evaluators were consistent. ICC values and significance were as follows: 0.01 ~ 0.20, slight agreement; 0.21 ~ 0.40, fair agreement, 0.41 ~ 0.60, moderate agreement; 0.61 ~ 0.80, significant agreement; 0.81 ~ 1.00, very good agreement. The statistical analysis software we applied was IBM SPSS Statistics version 26.0 (SPSS Inc., Chicago, IL, USA); a calculated result of *P* < 0.05 was considered statistically significant.

## Results

### Guideline characteristics

**Fig. 1:**
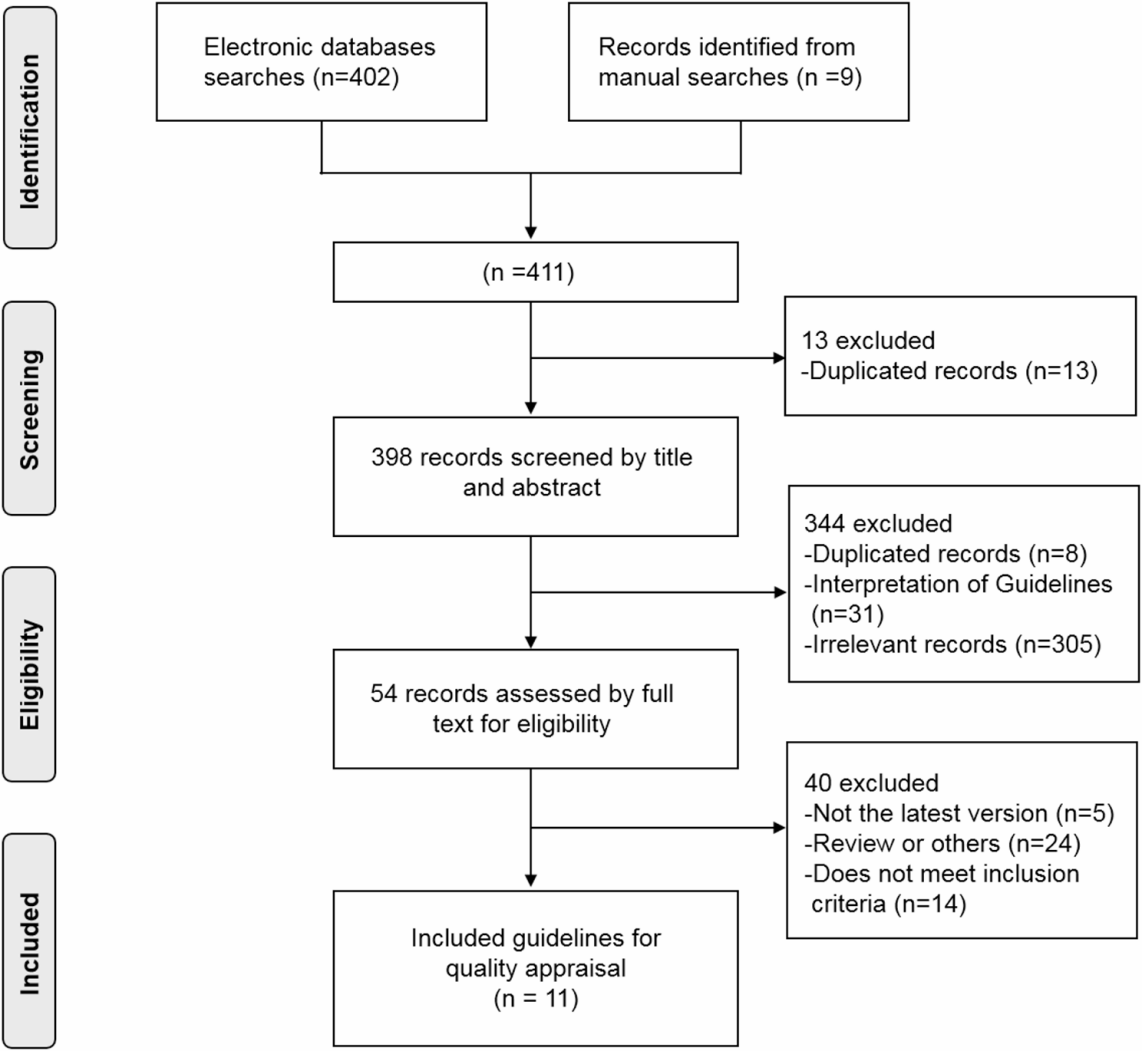
Flow chart of study selection

We systematically searched databases and websites according to the search strategy and reviewed the references to the guidelines, yielding a total of 411 records. We then screened 411 records according to the established inclusion and exclusion criteria. Eventually, 11 guidelines were selected for inclusion and full assessment (Fig. 1), and the main characteristics of each guideline are shown in Table 1. Of these, 2 guidelines were developed by international organizations or associations [13, 16], 2 guidelines were developed by organizations or associations in Japan [14, 15], 2 guidelines were developed by organizations or associations in China [19, 22], 1 guideline was developed by an organization or association in Germany [17], 1 guideline was developed by organizations or associations in Australia and New Zealand [18], 1 guideline was developed by the United Kingdom [20], and 2 guidelines were developed by international organizations for resource-limited settings [21, 23].

**Table 1:** Characteristics of the identified guidelines for the infection management of sepsis

Author/ organization	Short name	Year	Country applied	Version	Title	Funding	Grading system used	Development method
Evans L, et al. [13]	Ev	2021	International	Revised version	Surviving Sepsis Campaign: International Guidelines for Management of Sepsis and Septic Shock 2021	SCCM, ESICM	GRADE	EB
Egi M, et al. [14]	Eg	2021	Japan	Revised version	The Japanese Clinical Practice Guidelines for Management of Sepsis and Septic Shock 2020 (J-SSCG 2020)	JSICM, JAAM	GRADE	EB
JAID, JSC [15]	JA	2021	Japan	Revised version	The JAID/JSC guidelines for management of infectious diseases 2017 - Sepsis and catheter-related bloodstream infection	Not reported	Unclear	EB
Weiss SL, et al. [16]	We	2020	International	Original version	Surviving sepsis campaign international guidelines for the management of septic shock and sepsis-associated organ dysfunction in children	SCCM, ESICM	GRADE	EB
DGHO (AGIHO, iCHOP) [17]	DG	2019	Germany	Revised version	Management of sepsis in neutropenic cancer patients: 2018 guidelines from the Infectious Diseases Working Party (AGIHO) and Intensive Care Working Party (iCHOP) of the German Society of Hematology and Medical Oncology (DGHO)	Not reported	Unclear	EB
SOMANZ [18]	SO	2017	Australia, New Zealand	Original version	SOMANZ guidelines for the investigation and management sepsis in pregnancy	SOMANZ	GRADE	EB
CSCCM [19]	CS	2015	China	Original version	Chinese guidelines for management of severe sepsis and septic shock 2014	Not reported	GRADE	EB
NICE [20]	NI	2017	UK	Revised version	Sepsis: recognition, diagnosis and early management	NICE	GRADE	EB
Thwaites CL, et al. [21]	Th	2016	Resource–limited Settings	Original version	Recommendations for infection management in patients with sepsis and septic shock in resource-limited settings	Not reported	GRADE	EB
CCEP, CRHA [22]	CC	2018	China	Original version	Guidelines for the Emergency Management of Sepsis/Septic shock in China (2018)	Not reported	GRADE	EB
Dünser MW, et al. [23]	Dü	2012	Resource–limited Settings	Original version	Recommendations for sepsis management in resource-limited settings	Not reported	Unclear	EB

SCCM, Society of Critical Care Medicine; ESICM, European Society of Intensive Care Medicine; JSICM, Japanese Society of Intensive Care Medicine; JAAM, Japanese Association for Acute Medicine; DGHO, German Society of Hematology and Medical Oncology; AGIHO, Infectious Diseases Working Party; iCHOP, Intensive Care Working Party, SOMANZ, Society of Obstetric Medicine Australia and New Zealand; CAA, Chinese Association of Anesthesiologists; CSCCM, Chinese Society of Critical Care Medicine; NICE, National Institute for Health and Care Excellence; CCEP, Chinese College of Emergency Physicians; CRHA, Chinese Research Hospital Association; EB, evidence-based guideline

### Quality evaluation of the guidelines

The AGREE II instrument was used to assess the methodological quality of the final included guidelines, with the score results shown in Table 2. The mean scores for all guidelines across the six quality domains ranged from high to low as follows, with a scope and purpose score of 93.6% (range, 79.6–98.1%), clarity of presentation score of 91.4% (range, 64.8–98.1%), editorial independence score of 72.7% (range, 5.6–100.0%), stakeholder involvement score of 68.9% (range, 48.1–98.1%), rigor of development score of 67.6% (range, 43.1–86.1%), and applicability score of 64.8% (range, 51.4–76.4%). The overall evaluation scores of all guidelines were greater than 60%, and they were recommended for use. Of note, four guidelines scored greater than 60% in each quality domain and the final score was greater than 80%, which is a relatively desirable score [13, 14, 16, 20]. In this study, the ICCs of all three assessors who applied the AGREE II tool for scoring were greater than 0.8, which suggests very good agreement in item scores between raters.

**Fig. 2:**
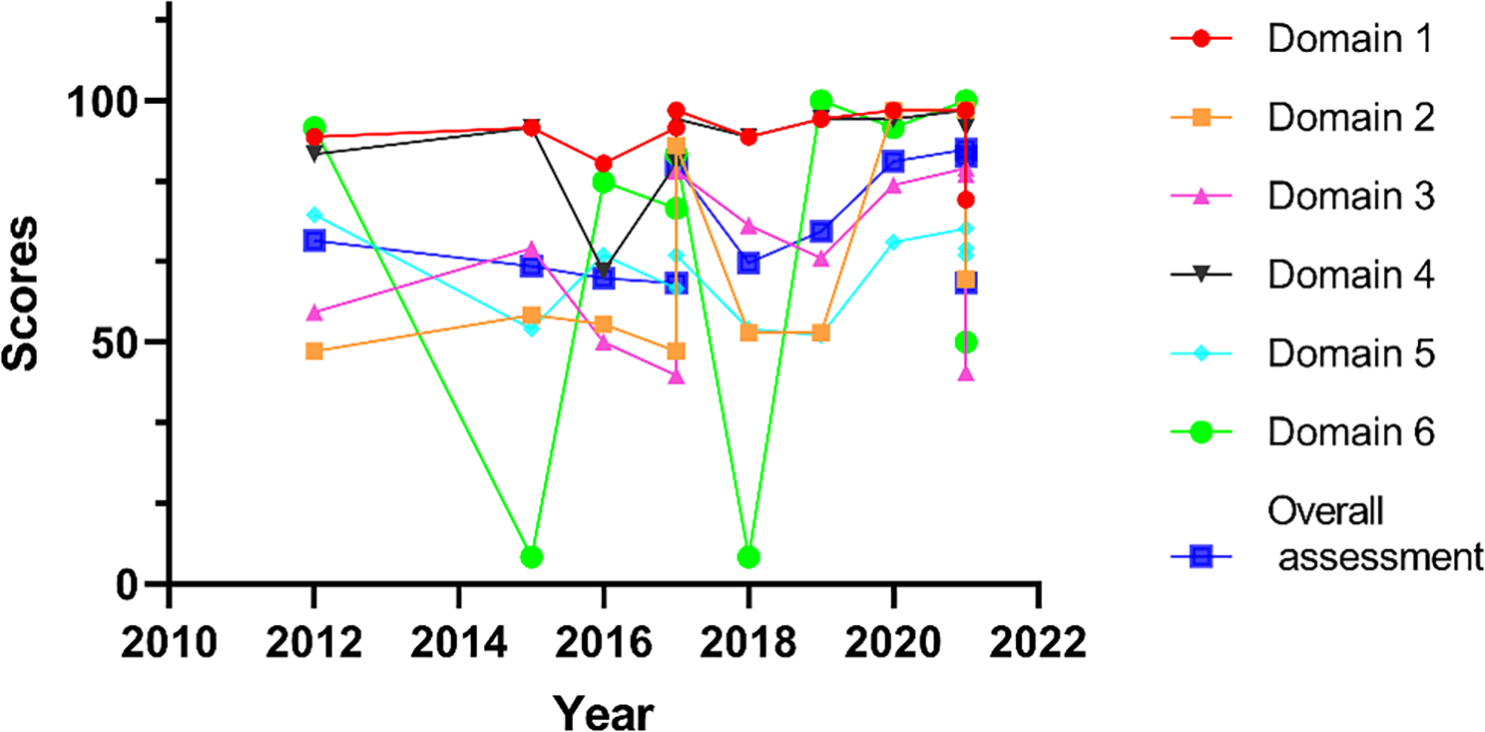
Distribution and trend of quality scores over time

The distribution of the six quality domain scores across the timeline for the included guidelines is shown in Fig. 2. With the passage of time, the quality scores of Domains 1, 4 are at a high level with a gentle trend; Domains 2, 3 show a significant upward trend; Domain 5 has average quality scores with no significant trend, and Domain 6 has the highest fluctuation and a very unstable trend. However, the overall assessment of the guidelines shows an upward trend in quality over time.

**Table 2:** Assessment of the quality of the included guidelines using AGREE II instrument

No.	Guideline	Scope and purpose	Stakeholder involvement	Rigour of development	Clarity of Presentation	Applicability	Editorial independence	ICC	Overall assessment
1	Evans L, et al. [13]	98.1%	98.1%	86.1%	98.1%	73.6%	100.0%	0.913	89.9% R
2	Egi M, et al. [14]	98.1%	98.1%	84.7%	96.3%	69.4%	100.0%	0.874	88.4% R
3	JAID, JSC [15]	79.6%	63.0%	43.8%	94.4%	68.1%	50.0%	0.929	62.3% R
4	Weiss SL, et al. [16]	98.1%	98.1%	82.6%	96.3%	70.8%	94.4%	0.850	87.4% R
5	DGHO(AGIHO, iCHOP) [17]	96.3%	51.9%	67.4%	96.3%	51.4%	100.0%	0.939	72.9% R
6	SOMANZ [18]	94.4%	48.1%	43.1%	87.0%	61.1%	77.8%	0.927	62.3% R
7	CSCCM [19]	94.4%	55.6%	69.4%	94.4%	52.8%	5.6%	0.949	65.7% R
8	NICE [20]	98.1%	90.7%	85.4%	96.3%	68.1%	88.9%	0.855	86.5% R
9	Thwaites CL, et al. [21]	87.0%	53.7%	50.0%	64.8%	68.1%	83.3%	0.959	63.3% R
10	CCEP, CRHA [22]	92.6%	51.9%	74.3%	92.6%	52.8%	5.6%	0.956	66.4% R
11	Dünser MW, et al. [23]	92.6%	48.1%	56.3%	88.9%	76.4%	94.4%	0.926	71.0% R
	mean scores (range)	93.6% (79.6–98.1%)	68.9% (48.1–98.1%)	67.6% (43.1–86.1%)	91.4% (64.8–98.1%)	64.8% (51.4–76.4%)	72.7% (5.6–100.0%)	—	—

R, recommended; RM, recommended with modifications; NR, not recommended

### Strength of recommendation and level of evidence

Of the 11 included clinical practice guidelines, 8 guidelines used the GRADE criteria [13, 14, 16, 18–22], and 3 guidelines graded the strength of recommendations and level of evidence, but did not explicitly label what grading system was used [15, 17, 23] (Table 1).

A total of 128 relevant recommendations for infection management of sepsis were extracted from 11 included guidelines. After uniformly grading the recommendation intensity and evidence level of the guidelines using GRADE criteria, strong recommendation accounted for 12.5%, weak recommendation accounted for 73.4%, and BPS items accounted for 14.1%. Except for two guidelines [16, 22], the remaining nine guidelines were consistent with the overall level, with weak recommendations accounting for the highest proportion. There was a significant difference in the level of evidence, with very low evidence quality accounting for the highest proportion (60.2%), followed by low evidence (28.1%) and moderate evidence (11.7%), which did not yield high quality evidence. With the exception of two guidelines [19, 22], the remaining nine guidelines were consistent with the overall level and all had the highest proportion of very low evidence quality. The above data are shown in Table 3.

**Table 3:** Distribution of strength of recommendations and level of evidence in sepsis infection management guidelines

	No. of Recommendations	Strength of Recommendation, No. (%)			Level of Evidence, No. (%)				
		Strong	Weak	BPS		High	Moderate	Low	Very Low
Evans L, et al. [13]	21	2 (9.52)	13 (61.9)	6 (28.6)	0		1 (6.7)	5 (33.3)	9 (60)
Egi M, et al. [14]	20	0	19 (95)	1 (5)	0		3 (15.8)	2 (10.5)	14 (73.7)
JAID, JSC [15]	19	1 (5.26)	18 (94.7)	0	0		2 (16.7)	3 (25)	7 (58.3)
Weiss SL, et al. [16]	12	1 (8.33)	4 (33.3)	7 (58.3)	0		0	1 (20)	4 (80)
DGHO(AGIHO, iCHOP) [17]	8	2 (25)	6 (75)	0	0		0	3 (37.5)	5 (62.5)
SOMANZ [18]	2	0	2 (100)	0	0		0	0	2 (100)
CSCCM [19]	11	4 (36.36)	7 (63.6)	0	0		2 (18.2)	7 (63.6)	2 (18.2)
NICE [20]	10	0	10 (100)	0	0		0	2 (20)	8 (80)
Thwaites CL, et al. [21]	10	3 (30)	7 (70)	0	0		1 (10)	2 (20)	7 (70)
CCEP, CRHA [22]	10	3 (30)	3 (30)	4 (40)	0		3 (50)	3 (50)	0
Dünser MW, et al. [23]	5	0	5 (100)	0	0		0	1 (20)	4 (80)
Total, No. (%)	128	16 (12.5)	94 (73.4)	18 (14.1)	0		12 (11.7)	29 (28.1)	62 (60.2)

## Discussion

### Principal findings

In this study, we used the AGREE II tool to evaluate the methodological quality of the guidelines for sepsis infection management. Overall, the quality scores of all included guidelines were satisfactory, with scores mainly distributed at a medium quality level and partially distributed at a high-quality level. Locally, we found that most guidelines had consistent scoring trends in the same field or item, that is, they were high or low. In addition, some guidelines scored extremely in a certain field or item, that is, they received the highest or lowest score. High-quality research evidence is very scarce in the included guidelines, and the strength of the recommended items made is mainly weak.

### Quality evaluation of guidelines by AGREE II

The included guidelines have a degree of commonality and specificity in scoring across different quality domains or items. Therefore, we systematically discuss the included guidelines in terms of six quality domains of the AGREE II instrument, including 23 scoring items, and make suggestions to improve the methodological quality of future sepsis guidelines.

Domain 1. Scope and Purpose. The average score for this domain was 93.6% and ranked first among the six quality domains. The results showed that guideline developers were largely free of problems in this area when developing sepsis guidelines. Although the evaluation results are nearly ideal, our guideline evaluation team still suggests that guideline developers be able to clearly articulate the overall goals, specific health problems, and target population of the guideline when developing the guideline to facilitate initial identification by guideline users.

Domain 2. Stakeholder Involvement. The mean score in this domain was 68.9%, ranging from 48.1 to 98.1%. The main reason for the medium overall score and wide range span is that the views and choices of the target population (patients, public, etc.) are not taken into account. However, all four guidelines scored more than 90% in this regard, providing us with a good paradigm by inviting septic patients or families, or collecting questions and opinions from the public [13, 14, 16, 20]. In clinical practice, it is the most ideal doctor-patient relationship to carry out work in a cooperative manner between clinical medical workers and patients. Therefore, our guideline evaluation team suggests that in addition to inviting patients and families, future guidelines can also be developed to invite target populations with certain sepsis expertise, such as medical staff or medical students who have had sepsis. In addition, it is also a good way to visit the ward for a status survey.

Domain 3. Rigour of Development. The average score for this domain was 67.6%, ranking penultimate among the six quality domains. Domain 3 includes eight scoring items, which are the most among the six quality domains, and are mainly related to the process of collecting and synthesizing evidence, the method of formulating recommendations, and the method of updating recommendations. Although the contents are complex, they are organized. Eleven of the included guidelines had the following quality issues about the rigor of their development, two guidelines did not search for evidence with a systematic approach and did not clearly state the criteria for selecting evidence [15, 18]; four guidelines did not link closely enough between recommendations and supporting evidence, and there were issues such as recommendations that were seriously inconsistent with evidence, recommendations had no evidence or no explanation of the circumstances without evidence [15, 17, 18, 23]; none of the five guidelines described information about updates [18, 19, 21–23]; and all the guidelines had problems that did not provide relevant and complete external review information prior to publication. However, four guidelines scored more than 80% in this domain, which is a good paradigm [13, 14, 16, 20]. Rigor is an important component of the guideline development process and an important criterion for determining whether the guideline is credible and whether users should adopt it. Therefore, our guideline evaluation team suggests that future sepsis guidelines be developed in strict accordance with the regulations of AGREE II instrument domain 3 and with reference to PRISMA-P specifications.

Domain 4. Clarity of Presentation. This domain had an average score of 91.4% and ranked second among the six quality domains. The scoring results showed that the quality of this area was relatively ideal, and there were few problems in this area during the guideline development process. However, our guideline evaluation team also recommended that the language, structure, and format of the guidelines still need to be fully considered in the future guideline development.

Domain 5. Applicability. The mean score in this domain was 64.8%, ranking last among the six quality domains; this result was consistent with most previous studies of guideline assessment by other scholars [31–34]. The main reason for the low applicability score is that most sepsis guidelines do not fully take into account the facilitating and hindering factors in the application of the guidelines, as well as potential resource investment issues. For example, language restriction, regional epidemiological differences, the level of medical skills, the allocation of medical facilities, the need for guideline recommendation in clinical practice, the ability of patients and regions to pay for medical needs, expenditure and cost are a series of issues. In addition, four guidelines still had significant deficiencies in how suggests and/or companion tools were applied to practice [19, 21–23]. Therefore, our guideline evaluation team suggests that guideline developers must value issues related to applicability such as possible barriers and enablers to implementation, strategies to improve uptake, and resource implications of applying guidelines, as this may be key to improving guideline quality to a higher level. For example, to strengthen international cooperation, developers of guidelines should not only come from developed countries, but should also contain people from resource-limited areas, preferably including people with medical professional backgrounds in countries around the world.

Domain 6. Editorial Independence. The average score for this domain was 72.7%, which is a good score; however, it ranged from 5.6 to 100.0%, which was the largest of the six quality domains. In this study, three guidelines did not mention whether there was support from funds or sponsors [15, 19, 22], and we could not judge whether the development of guidelines was influenced by the views or interests of funding agencies, and therefore, we gave a very low score. The two guidelines did not mention or document whether there was a conflict of interest between members of the guideline development panel, and therefore, we gave a very low score in this quality item [19, 22]. However, we found that although the two guidelines were excellent in content, the overall score was low due to lack of standardized writing and very low scores in this domain; if the content in this domain was further standardized and refined, the score would be expected to reach 80% and become a high-quality guideline [19, 22]. Editorial independence aims at verifying the fairness and objectivity of the guidelines and precluding the impact of commercial or personal interests on the overall applicability and effectiveness of the guidelines. Therefore, our guideline evaluation team suggests that when developing the guideline, it is important to focus on interest issues, make detailed records, and try to stay as far away from competing interests as possible.

### Recommendation and level of evidence

Although the overall quality of infection management guidelines for sepsis has been trending upward over time, the guidelines are still dominated by weak recommendations, the quality of evidence remains predominantly low and very low quality, and there is no high-quality research evidence. Improvements in the overall quality of sepsis infection management guidelines should rely more on the generation of high-quality clinical research evidence, in addition to increased transparency and methodological rigor. It is noteworthy that most of the recommended items made are weakly recommended due to the fact that most of the relevant research evidence is of low and very low quality with significant uncertainty. This finding constitutes a major obstacle to the development of guidelines for the management of sepsis infections, as the evidence is mostly of low quality and highlights the gap between clinical practice evidence and current medical research.

The International Surviving Sepsis Campaign Guidelines (SSCG) [13] and the Japanese Sepsis Guidelines (J-SSCG) [14], which are high-impact guidelines developed in recent years, were the two guidelines with the highest scores in this quality assessment. The infection management recommendation items in the two guidelines were dominated by weak recommendations, and the quality of evidence was dominated by low and very low quality, which is a side effect of the fact that high-quality research evidence is very scarce. Regarding the content of the recommended items in this included guideline, there were only minor differences in the timing of antimicrobials, source control, antifungal therapy, antibiotic de-escalation, and biomarkers for discontinuation of antibiotics for sepsis infection management. However, there was greater heterogeneity in biomarkers for initiating antibiotics, personalization of infection source control, antimicrobial selection, antiviral therapy, pharmacodynamic/pharmacodynamic principles, and duration of antibiotics. The main reasons for the heterogeneity include the intricacies of sepsis itself, different epidemiologic characteristics and antimicrobial profiles of infections, inconsistencies in the target contextual issues, the existence of formulators’ preferences, irrational citation of research evidence, and the lack of high-quality supporting evidence. In addition, the fairness and tendency of guidelines to select evidence for different sepsis patients is a potential cause of heterogeneity in sepsis infection management programs. The best way to address the above knowledge gaps in order to improve the overall quality of guidelines and better guide clinical practice remains to improve the quality of evidence. Therefore, larger, multicenter, well-designed, adequately powered, population subgroup-specific randomized controlled trial studies on sepsis infection management are needed to reduce the knowledge gaps that limit evidence-based practice and to make more accurate and higher-level recommendations.

### Strengths and limitations of the study

The strengths of this study are as follows: (1) This study assessed guidelines from different quality domains, and each quality domain occupied an appropriate proportion of the weight, so the reliability of guideline assessment results is high. (2) GRADE method is used to analyze the recommendation strength and evidence level of guidelines, which helps to identify gaps in practice and gives guideline developers and readers a clearer picture of the evidence cited in the guidelines. (3) It provides a reference for clinical practitioners to select appropriate guidelines and useful recommended items, and provides guideline developers with ideas for updating and improving them.

Limitations of this study are as follows: (1) We only included guidelines published in English and Chinese languages and did not evaluate guidelines published in other languages. (2) AGREE II tools are mainly assessed in terms of the methodological quality of the guidelines, lack the ability to interpret the contents of the guidelines, and are unable to assess the impact of the guidelines on clinical practice. (3) The septic infection management guidelines were included in nearly a decade, with a large time span, during which the definition of sepsis terms, some research evidence and treatment concepts were updated, which somewhat increased heterogeneity among the guidelines.

## Conclusion

Guidelines for sepsis infection management vary in quality, but overall quality levels are satisfactory. In quality domains, there is still much room for improvement in the field of application, and three areas such as stakeholder involvement, rigour of development, and editorial independence need to be further improved. High-quality research evidence in the guidelines is very scarce, recommendations are variable, and more high-quality randomized controlled trial studies are needed to reduce the knowledge gaps that limit evidence-based practice and to make higher-level recommendations. It is recommended that guideline developers be oriented to address the issues of guideline quality and knowledge gaps in the management of sepsis infections in order to develop high-quality guidelines.

## Electronic supplementary material

Below is the link to the electronic supplementary material.

Supplementary Material 1

Supplementary Material 2

## Data Availability

All authors agree to share data from this review, which are available by contacting the corresponding author. E-mail: liuwenjun@kmmu.edu.cn.
