# Supplementary material for: Quality of guidelines for infection management in sepsis: a critical appraisal using the AGREE II instrument
Source: BMC Med Res Methodol. 2025 Feb 21;25:48. doi: 10.1186/s12874-025-02491-8 (PMC11846407; doi:10.1186/s12874-025-02491-8)
Supplement: Supplementary file 1 — Supplementary Material 1 [file 12874_2025_2491_MOESM1_ESM.pdf]

Appendix 1. Domains and items of the AGREE II instrument*

| Domain | Item | Content |
|---|---|---|
| 1. Scope and Purpose | 1 | The overall objective(s) of the guideline is (are) specifically described. |
| | 2 | The health question(s) covered by the guideline is (are) specifically described. |
| | 3 | The population (patients, public, etc.) to whom the guideline is meant to apply is specifically described. |
| 2. Stakeholder Involvement | 4 | The guideline development group includes individuals from all relevant professional groups. |
| | 5 | The views and preferences of the target population (patients, public, etc.) have been sought. |
| | 6 | The target users of the guideline are clearly defined. |
| 3. Rigour of Development | 7 | Systematic methods were used to search for evidence. |
| | 8 | The criteria for selecting the evidence are clearly described. |
| | 9 | The strengths and limitations of the body of evidence are clearly described. |
| | 10 | The methods for formulating the recommendations are clearly described. |
| | 11 | The health benefits, side effects, and risks have been considered in formulating the recommendations. |
| | 12 | There is an explicit link between the recommendations and the supporting evidence. |
| | 13 | The guideline has been externally reviewed by experts prior to its publication. |
| | 14 | A procedure for updating the guideline is provided. |
| 4. Clarity of Presentation | 15 | The recommendations are specific and unambiguous. |
| | 16 | The different options for management of the condition or health issue are clearly presented. |
| | 17 | Key recommendations are easily identifiable. |
| 5. Applicability | 18 | The guideline describes facilitators and barriers to its application. |
| | 19 | The guideline provides advice and/or tools on how the recommendations can be put into practice. |
| | 20 | The potential resource implications of applying the recommendations have been considered. |
| | 21 | The guideline presents monitoring and/or auditing criteria. |
| 6. Editorial Independence | 22 | The views of the funding body have not influenced the content of the guideline. |
| | 23 | Competing interests of guideline development group members have been recorded and addressed. |

* References: [25].
