# Supplementary material for: Quality of guidelines for infection management in sepsis: a critical appraisal using the AGREE II instrument
Source: BMC Med Res Methodol. 2025 Feb 21;25:48. doi: 10.1186/s12874-025-02491-8 (PMC11846407; doi:10.1186/s12874-025-02491-8)
Supplement: Supplementary file 2 — Supplementary Material 2 [file 12874_2025_2491_MOESM2_ESM.pdf]

Additional file 2. Grading of Recommendations, Assessment, Development and Evaluation (GRADE) approach

➢ Quality of Evidence Grades

Table S3. Definition of evidence quality grades

Grade	Definition
**High**	We are very confident that the true effect lies close to that of the estimate of the effect.
**Moderate**	We are moderately confident in the effect estimate: The true effect is likely to be close to the estimate of the effect, but there is a possibility that it is substantially different.
**Low**	Our confidence in the effect estimate is limited: The true effect may be substantially different from the estimate of the effect.
**Very Low**	We have very little confidence in the effect estimate: The true effect is likely to be substantially different from the estimate of effect.

Table S4. Underlying methodology for determining the quality of evidence

Grade	description
**High**	RCTs
**Moderate**	Downgraded RCTs or upgraded observational studies
**Low**	Well-done observational studies with RCTs
**Very Low**	Downgraded controlled studies or expert opinion or other evidence

Table S5. Factors that can reduce the quality of the evidence

Factor	Consequence
**Limitations in study design or execution (risk of bias)**	↓ 1 or 2 levels
**Inconsistency of results**	↓ 1 or 2 levels
**Indirectness of evidence**	↓ 1 or 2 levels
**Imprecision**	↓ 1 or 2 levels
**Publication bias**	↓ 1 or 2 levels

Table S6. Factors that can increase the quality of the evidence

Factor	Consequence
**Large magnitude of effect**	↑ 1 or 2 levels
**All plausible confounding would reduce the demonstrated effect or increase the effect if no effect was observed**	↑ 1 level
**Dose-response gradient**	↑ 1 level

➢ Recommendations and their strength

Table S7. Definition of recommendations and their strength

Recommendation	Definition
**Strong Recommendation**	The desirable effects of an intervention outweigh its undesirable effects (strong recommendation for an intervention) or that the undesirable effects of an intervention outweigh its desirable effects (strong recommendation against an intervention).
**Weak Recommendation**	The desirable effects probably outweigh the undesirable effects (weak recommendation for an intervention) or undesirable effects probably outweigh the desirable effects (weak recommendation against an intervention) but appreciable uncertainty exists.

Table S8. Implications of strong and weak recommendations for different users of guidelines

	Strong Recommendation	Weak Recommendation
For patients	Most individuals in this situation would want the recommended course of action and only a small proportion would not.	The majority of individuals in this situation would want the suggested course of action, but many would not.
For clinicians	Most individuals should receive the recommended course of action. Adherence to this recommendation according to the guideline could be used as a quality criterion or performance indicator. Formal decision aids are not likely to be needed to help individuals make decisions consistent with their values and preferences.	Recognize that different choices will be appropriate for different patients, and that you must help each patient arrive at a management decision consistent with her or his values and preferences. Decision aids may well be useful helping individuals making decisions consistent with their values and preferences. Clinicians should expect to spend more time with patients when working towards a decision.
For policy makers	The recommendation can be adapted as policy in most situations including for the use as performance indicators.	Policy making will require substantial debates and involvement of many stakeholders. Policies are also more likely to vary between regions. Performance indicators would have to focus on the fact that adequate deliberation about the management options has taken place.

Table S9. Domains that contribute to the strength of a recommendation

Domain	Comment
Balance between desirable and undesirable outcomes (trade-offs) taking into account:<br>- best estimates of the magnitude of effects on desirable and undesirable outcomes<br>- importance of outcomes (estimated typical values and preferences)	The larger the differences between the desirable and undesirable consequences, the more likely a strong recommendation is warranted. The smaller the net benefit and the lower certainty for that benefit, the more likely a weak recommendation is warranted

Confidence in the magnitude of estimates of effect of the interventions on important outcomes (overall quality of evidence for outcomes)	The higher the quality of evidence, the more likely a strong recommendation is warranted
Confidence in values and preferences and their variability	The greater the variability in values and preferences, or uncertainty about typical values and preferences, the more likely a weak recommendation is warranted
Resource use	The higher the costs of an intervention (the more resources consumed), the less likely a strong recommendation is warranted

Notes: The above evidence grading and recommendation strength methods refer to the GRADE Handbook developed by the Grading of Recommendations, Assessment, Development and Evaluation (GRADE) Working Group (www.gradeworkinggroup.org) [29].
